# White-Tailed Deer Serum Kills the Lyme Disease Spirochete, *Borrelia burgdorferi*

**DOI:** 10.1089/vbz.2022.0095

**Published:** 2023-05-12

**Authors:** Patrick Pearson, Connor Rich, Martin J.R. Feehan, Stephen S. Ditchkoff, Stephen M. Rich

**Affiliations:** ^1^ Laboratory of Medical Zoology, Department of Microbiology, University of Massachusetts, Amherst, Massachusetts, USA.; ^2^ Massachusetts Division of Fisheries and Wildlife, Westborough, Massachusetts, USA.; ^3^ College of Forestry, Wildlife and Environment, Auburn University, Auburn, Alabama, USA.

**Keywords:** *Borrelia burgdorferi*, white-tailed deer, serum, reservoir competence

## Abstract

is a human pathogen vectored by *Ixodes* ticks and maintained in nature by a suite of competent vertebrate reservoirs. White-tailed deer (WTD) are considered to be noncompetent reservoirs for *B. burgdorferi*. Sera from other deer species have been found to be borreliacidal, and similar mechanisms could explain the lack of reservoir competence of WTD. Therefore, we determined whether WTD serum can kill *B. burgdorferi.* Using an *in vitro* serum sensitivity assay and subculturing of spirochetes, we demonstrated that WTD serum effectively kills *B. burgdorferi*. The borreliacidal activity of WTD serum likely contributes to the inability of WTD to efficiently harbor and transmit *B. burgdorferi*.

## Introduction

*B**orrelia burgdorferi sensu* *stricto* (hereafter *B. burgdorferi*) is the causative agent of Lyme disease in the United States and circulates in nature between *Ixodes* ticks and vertebrate hosts (Steere et al., 2016). However, not all vertebrate host species fed upon by *Ixodes* ticks serve as competent reservoirs for *B. burgdorferi*. White-tailed deer (WTD) (*Odocoileus virginianus*) are considered reservoir incompetent, since they are generally resistant to maintaining a *B. burgdorferi* infection, and fail to infect feeding ticks (Luttrell et al., 1994; Telford et al., 1988).

The ability of *B. burgdorferi* to infect a potential host species in nature is typically correlated with the ability of *B. burgdorferi* to survive in that species' serum in the laboratory (Lin et al., 2020). It has been previously demonstrated that serum from other deer species is borreliacidal, and this is most likely due to the effects of complement, a component of host innate immunity (Bhide et al., 2005; Kurtenbach et al., 1998; Nelson et al., 2000; Ullmann et al., 2003). However, to our knowledge, the ability of WTD serum to kill *B. burgdorferi* has not yet been evaluated. Therefore, we determined whether WTD serum has the capacity to kill different *B. burgdorferi* strains.

## Materials and Methods

Low-passage clonal strains of *B. burgdorferi* were isolated directly from field-collected adult *Ixodes scapularis* ticks, by limiting dilution. The strains were genotyped based on their outer surface protein C (*ospC*) sequence using Luminex technology as previously described (Pearson et al., 2022) and by Sanger sequencing. No mixed *ospC* sequences were detected by either method. *B. burgdorferi* B31 clone 5A1 (*ospC* type A) was purchased from BEI resources.

Serum was collected from WTD (*N* = 2) belonging to a semi-captive deer herd at Auburn University, Alabama (Newbolt et al., 2017). WTD were chosen from this area since there is low transmission of *B. burgdorferi* locally and thus low risk of confounding anti-*B. burgdorferi* antibodies (Fleshman et al., 2022; Murdock et al., 2009). A modified serum sensitivity assay was used to determine the impact of WTD serum on *B. burgdorferi* (Lin et al., 2022). In brief, the strains were grown to mid-logarithmic phase in BSK-H media with 6% rabbit serum (BSK-H complete; Sigma) and diluted to 3 × 10^6^ cells/mL in BSK-H media without rabbit serum (BSK-H incomplete; Sigma).

An equal volume of WTD serum was mixed with the culture (50% WTD serum final concentration), the mixture was split into triplicate wells on a 96-well plate, and incubated at 34°C for 4 h. BSK-H incomplete media was substituted for WTD serum as a control. We also tested the sensitivity of one strain in BSK-H incomplete media supplemented with heat-inactivated (56°C for 30 min) WTD serum. Owing to the low volume of WTD serum only one strain could be incubated in heat-inactivated serum. The number of viable cells were counted by darkfield microscopy at 0 and 4 h.

Viability was defined as motility and retention of characteristic spirochete shape (Nelson et al., 2000). Percent survival was calculated as the number of viable cells after 4 h over the number of viable cells at the 0-h time point multiplied by 100. This value was then normalized to the percent survival of the cells in the control group. To confirm borreliacidal activity by WTD serum observed during the serum sensitivity assay, an aliquot from the control and test samples in the 96-well plate at the 4-h time point was passed to BSK-H complete media in 1.5 mL tubes and incubated at 34°C.

Following the method of Nelson et al. (2000), the subculture tubes were incubated for 6–8 days and checked by darkfield microscopy for the presence of *B. burgdorferi* growth. Detection of antibodies specific for *B. burgdorferi* in the WTD sera was determined retrospectively using the Idexx SNAP 4Dx Plus kit (Murdock et al., 2009). Serum from only one of the two WTD (deer 2, Table 1) was tested for antibodies and was determined to be negative. The sample volume for the second deer was insufficient to test for antibodies (deer 1, Table 1).

**Table 1.** White-Tailed Deer Serum Is Borreliacidal for Different *Borrelia burgdorferi* Strains

WTD serum	Borrelia burgdorferi strain	% Survival	Growth detected in subculture (days 6–8)^a^
Deer 1	5A1	0	No
	*ospC* L	0	No
	*ospC* O	0	No
Deer 2	*ospC* I	0	No
	*ospC* A	0	No
	*ospC* M	0	No
	*ospC* G	0	No
	*ospC* H	0	No
Deer 2 (HI)	*ospC* H	34	Yes

Survival of different *B. burgdorferi* strains was tested using WTD sera collected from two individuals. Mean percent survival calculated from three replicates.

^a^ All subcultures were checked for *B. burgdorferi* growth on days 6–8 and a subset was also checked weeks later (see Results section).

HI, heat inactivated; WTD, white-tailed deer.

### Ethical statement

All procedures handling animals were approved by Auburn University IACUC (PRN no. 2019-3599).

## Results

The WTD deer sera were borreliacidal to all *B. burgdorferi* strains tested (Table 1). No viable spirochetes were observed at 4 h by darkfield microscopy for any *B. burgdorferi* strains incubated with WTD sera collected from two individuals. The subculture tubes checked on days 6–8 had no growth for all *B. burgdorferi* strains previously incubated with WTD serum during the serum sensitivity assay. As routine practice, we checked some old subcultures for contaminant growth weeks later.

Surprisingly, *B. burgdorferi* growth was observed in at least one of the replicate subculture tubes for the *B. burgdorferi ospC* type O, I, and G strains. All subcultures were checked weeks later except those from the experiments using the *ospC* type A, H, and M strains since they had already been discarded. For the control groups in the serum sensitivity assay, percent survival was normalized to 100% and growth was observed in all subcultures. When heat-inactivated WTD serum was tested against the *B. burgdorferi ospC* type H strain, percent survival was 34% and growth was observed in the subculture (Table 1).

## Discussion

We report that WTD serum can effectively kill genetically distinct *B. burgdorferi* strains, as defined by their *ospC* sequence. Strikingly, no viable spirochetes were observed at the end of the 4-h serum sensitivity assay for any *B. burgdorferi* strains tested. The effective killing of spirochetes was confirmed by a lack of growth of the strains when passaged to fresh BSK-H complete media and checked during days 6–8. However, growth was observed in subcultures for three of the strains when checked weeks later. This suggests that a very small population of spirochetes from these *B. burgdorferi* strains survived in the presence of WTD serum, but that it takes several weeks to appear in subculture. These surviving spirochetes were most likely missed at the end of the serum sensitivity assay due to their low numbers. It is possible that spirochetes would have also grown in subculture for the *ospC* type A, M, and H strains previously incubated in WTD serum, but we were not able to confirm at later dates.

Interestingly, 34% of spirochetes from the *ospC* type H strain survived incubation with heat-inactivated WTD serum and grew in subculture (Table 1). This suggests that the killing of *B. burgdorferi* by WTD serum may be due in part to the effects of complement. In similar experiments, heat-inactivation of serum typically results in the complete, or near-complete, loss of borreliacidal activity (Kurtenbach et al., 1998; Nelson et al., 2000; Ullmann et al., 2003). It is unclear why survival in heat-inactivated serum was not closer to 100% in this experiment.

This may be artifactual due to a clumping of spirochetes observed after 4 h of incubation with heat-inactivated WTD serum. Clumping confounds the ability to quantify the number of single planktonic spirochetes. Future experiments should confirm this result and determine whether complement-independent factors are also responsible for the borreliacidal activity of WTD serum. Although we could not test the serum from both WTD used in the experiments, antibody-mediated killing of spirochetes is unlikely since the available serum tested was negative for anti-*B. burgdorferi* antibodies and given the very low prevalence of *B. burgdorferi* infected ticks in Alabama (Fleshman et al., 2022).

Prior studies have demonstrated that sera collected from red, sika, mule, fallow, and roe deer kill different *Borrelia* genospecies (Bhide et al., 2005; Kurtenbach et al., 1998; Nelson et al., 2000; Ullmann et al., 2003), but no study has previously tested WTD serum. Our results add to this growing list and demonstrate that WTD serum is borreliacidal for different *B. burgdorferi* strains. WTD serum displays potent killing activity against *B. burgdorferi* and is likely a mechanism contributing to the reservoir incompetence of WTD.
